# Adoption of digital twins as a sustainable energy solution: Determinants to adoption in household

**DOI:** 10.1016/j.heliyon.2024.e25782

**Published:** 2024-02-07

**Authors:** Joana Coelho, Tiago Oliveira, Catarina Neves, Stylianos Karatzas

**Affiliations:** a NOVA Information Management School (NOVA IMS), Universidade Nova de Lisboa, Campus de Campolide, 1070-312, Lisboa, Portugal; b University of Patras, University Campus, 265 04, Rio, Patras, Greece

**Keywords:** Digital twin, Sustainable energy solutions, Technology adoption, Mixed-methods

## Abstract

Digital Twin (DT) consists of a recent technology that can enable sustainability. However, Digital Twins are still in early stages of adoption, especially in households, and so the determinants to this adoption have not yet been determined. The aim of this study is to fill this research gap through providing a conceptual model of the drivers to the adoption of Digital Twins in households and it's relation to well-being. This study is developed as a mixed-methods research. The model is produced qualitatively, based on literature discoveries and key findings from interviews with experts and possible consumers. Afterwards, the model was validated with data collected through a questionnaire with 149 respondents. Results show that a set of informational, social, environmental and utility factors can influence the intention to adopt Digital Twins as a sustainable energy solution, and consequently the perceived well-being.

## 1 Introduction

Nowadays sustainability has become an imperative topic. As climate change effects are starting to be evident, numerous people are turning to the adoption of sustainable lifestyle alternatives. One of the areas that is registering this change is the energy sector, as consumers are starting to opt for the adoption of sustainable energy alternatives in their homes [1]. However, 70 % of CO2 emissions still originate from households [2] and urban areas contribute to most global energy consumption and carbon emissions [3]. In the effort towards energy conservation, consumers can be major actors by adopting innovative solutions [4]. Policy makers are also acting towards ensuring a sustainable future. This study is developed in line with the concretization of “Goal 7: Ensure access to affordable, reliable, sustainable and modern energy for all” from the United Nations 17 Sustainable Development Goals (SDGs) [5]. However, studies have proven that establishing policies is insufficient to drive the adoption of green energy [6,7]. In fact, countries should be taking a joint approach to the achievement of the SDGs, as the improvement of environmental factors of only one or limited countries is unlikely to generalize environmental sustainability across the globe [8].

Digital Twin (DT) is an emerging technology that sustains digital transformation by supporting new business models [9]. A DT provides a virtual representation of a physical system, which is constantly updated through information collection [9]. This technology has potential to enhance the sustainability of buildings through enabling smart electricity consumption [[10], [11], [12]]. However, current literature lacks an account of the determinants to the adoption of DTs in households and overlooks how the technology can contribute to well-being. In fact, most studies have focused on the technological and insfrastructural side and requirements for the implementation of this solutions, overlooking the consumer perspective. Therefore, by employing a mixed-methods approach, this paper aims to fill these research gaps through achieving the following research objective: determine the drivers to the adoption of DTs as a sustainable energy solution in households.

This study presents a twofold contribution to literature. Firstly, it delivers a model of the determinants to the adoption of DTs in households. This model is not only relevant for literature and research purposes, but also to energy companies that may be interested in selling DTs as a service. This model allows to understand consumer behavior and thus employ calculated marketing and policy strategies to better target the public. Secondly, this article establishes the connection of DTs and well-being through specifying the factors that enable it when applied to energy in households.

This research paper is structured as follows. Section 2 provides theoretical background to the topics of DTs and sustainable energy adoption. Section 3 elaborates on the interviews conducted as part of the qualitative study. Section 4 describes the research model and hypothesis developed. Section 5 explains the methodology employed in the quantitative study as well as the collected data. Section 6 elaborates on the results obtained. Section 7 presents the key learnings from this study, along with further research recommendations. Finally, Section 8 draws the conclusions that can be taken from this paper.

## 2 Theoretical background

### 2.1 Digital twin definition

The concept of Digital Twin was first introduced by Michael Grieves in 2003 at a presentation on product life-cycle management [13]. Since then, the concept has constantly evolved as the interest on the technology grew [14]. DTs are experiencing a fast development, as researchers are starting to investigate its numerous applications [15]. This phenomenon led to multiple definitions and characterizations of the technology. In a recent study, VanDerHorn & Mahadevan reviewed 46 different definitions of DT, aiming to formulte the most accurate and complete definition of the concept. Their study resulted in a generalized definition of Digital Twin as “*a virtual representation of a physical system (and its associated environment and processes) that is updated through the exchange of information between the physical and virtual systems*” [9]. Moreover, a DT is characterized by three main components: (1) the physical entity and reality, (2) the virtual representation, and (3) the interconnected data exchanging information between the physical and virtual realities [13,16]. As a constantly-updated and realistic reproduction of a physical system, DTs can optimize the physical environment, relying on models generated from collected data [17]. Through producing machine learning models and data visualizations based on historical energy behavior patterns and short/long-term energy forecasting, DTs can provide the energy profile of a consumer [4,18]. Moreover, DTs have numerous applications as it does not consist of a specific technology, but rather a flexible concept implemented through the integration of various technologies [19]. These can be divided into data related technologies, high-fidelity modelling technologies and model-based simulation technologies [15]. The integration of these technologies, along with their lack of global standards of performance, leads to concerns regarding data and system security, which can be a challenge to the adoption of DTs [19]. Other than technical challenges, DTs can also present cultural barriers to adoption, which require disrupting current practices [9].

### 2.2 Digital twin as a sustainable energy solution

Regarding its application to energy services, DTs can operate as a recommendation system. In this scenario, DTs allow for data analysis of the energy consumption, and provide economic, technical or social recommendations to the end-consumer [4] and even forecast the residential energy load demand [20]. Thus, DTs allow to achieve sustainability and financial objectives [11]. In Italy, a pilot DT has been implemented at a building of the University of Brescia to test the technology in a real environment, with the aim of assessing the sustainability of the building [21]. This testing infrastructure provides proof that DTs contribute to sustainable and reliable energy systems [22]. DTs can contribute to reduce and rationalize energy consumption through the use of IoT-based control systems [10]. Riedelsheimer et al. (2021) proposes a DT V-Model, which consists of a methodology to develop DTs of IoT-based products with the aim of optimizing the systems sustainability. This model defines the main DT properties for energy saving practices, which are monitoring and analysing energy consumption; comparing planned and actual energy consumption; and recommending energy optimization practices, improving decision making [12]. Given its strong benefits, DTs can be considered as a sustainable energy solution. In fact, prior research on the topic has highlighted the role of DTs in the energy area, as DTs can be applied to the whole energy grid, digitalizing all elements, services and events of the grid [23]. To achieve the fullest potential of DTs, a set of key technologies, mainly IoT, are needed, allowing energy comsumption analysis and forecast, and energy management and optimization services [24]. Given this, we decided to focus on DTs as a sustainable energy solution.

### 2.3 Sustainable energy services adoption

The adoption of innovative energy services is on upward trend [4]. Recent research suggests a model of sustainable technology adoption in the residential sector (STARS) which defines the intention to adopt sustainable technology as being influenced by a set of motivational, household demographics, electricity-consumption related, privacy related and innovation related variables [1]. Another author summarizes the determinants to the adoption of green energy into four categories: economic factors; social psychological factors; factors related to national culture and factors related to the environment [25]. According to the same study, the consumer's pursuit for well-being can significantly influence green energy adoption [25]. However, the concept of DT related to well-being is still on an early stage of research, as this is an emerging field when compared to the DTs main applications [26].

When studying the consumers' intention to adopt green energy, it is important to understand the willingness to pay for the service [25]. As the adoption of sustainable energy services is still in an early stage, and it is subject to financial factors and lack of support from governments [4], willingness to pay can be fairly low [27]. However, some determinants have a positive impact on willingness to pay for green energy, namely acceptance of green energy, social norms and moral obligations and knowledge about green energy [28]. Brand attitude and purchase are also predictors of intention to adopt green energy. Factors such as the perceived utilitarian benefits, warm glow and environmental concern can improve a consumer's attitude towards green energy brands and consequently increase the purchase intention [29].

Regarding the consumers’ willingness to change household appliances for a more energy efficient alternative, the public is more tempted to adopt an energy-efficient appliance if there is social influence, if the consumer is concerned about the environment, if the equipment has a green energy label, providing energy efficiency and money savings, and if the technology is present on organizational and web media channels. However, operation and maintenance have proven to have a negative effect on adoption, unless the individual is concerned about the environment [30,31].

Table 1 summarizes the determinants of sustainable energy solutions adoption that were tested in prior research. To obtain this table, the explanatory variables identified in each article were recorded. The most relevant and frequent ones were then presented on Table 1 as columns. The crosses identify all works that have tested them as determinants of adoption. Having collected all necessary information from literature, we proceeded to perform qualitative research to further explore possible adoption drivers of DTs.

**Table 1:** Summary of determinants derived from theoretical background.

Source	Adoption determinants											
	Green self-identity	Knowledge	Perceived privacy risks	Perceived value	Perceived wellbeing	Social influence	Comfort sensation	Household composition	Electricity consumption	Economic factors	Culture factors	Effort
[22]			X									
[1]	X		X	X				X	X			
[25]	X	X		X	X	X				X	X	
[27]	X	X				X						
[28]	X	X				X						
[29]	X			X	X							
[30]						X	X			X		X
[31]	X						X			X		X

## 3 Mixed-methods approach

Due to the lack of widespread information regarding the DT topic, we have opted for a mixed-methods approach, conducting both qualitative and quantitative studies, as only one method might not be sufficient for complete results. The mixed-methods approach allows to converge findings to provide stronger evidence, increasing the generalizability of results and consequently presenting more complete knowledge [32]. The purpose of the mixed methods is therefore developmental, since the results of the qualitative study will be used to develop the research model, that will be later tested on the quantitative study. This approach allows us to overcome the weaknesses of each method [33]. While the qualitative study may present a rather small sample, and generalization of results may be difficult, the purpose of this method is to identify possible determinants of DT use. Later, to provide greater confidence on the results, the model is tested statistically with quantitative data. Thus, all guidelines provided by Ref. [33] were followed.

### 3.1 Qualitative study methodology

As part of the investigative process, we conducted a qualitative study. Given the generally limited knowledge on the topic, a purposive sampling was used for the qualitative study (and a probability sampling for the quantitative study). In qualitative study, interviewees were selected within a university staff and students. The use of purposive sample for the qualitative study is common, especially on energy-related topics [34,35]. Therefore, 15 interviews were conducted within a Portugal university. These interviews aim to understand the public's standpoint regarding digital twins. The interviewees' sample consisted of experts on the topic and consumers that could be possible adopters of DTs. From the 15 interviews conducted, 13 were consumers and 2 were experts. Through interviewing both specialists and the general public, we gathered different opinions and drivers to the adoption of DTs. The interviewees were mainly decision-makers regarding the adoption of new technologies in their household and represent various household types. An introductory text and image explaining the DT concept was presenting in the beginning of the interviews. See **Appendix A** for interviewees' details. All interviewees admitted being willing to adopt a DT in the future.

All interviews were conducted in English and recorded exclusively for transcription purposes. On the beginning of the interview, the participant was contextualized on the topic of DTs. Afterwards, an interview guide was followed to conduct the interview questions. See **Appendix B** for the interview guide. On average, interviews lasted for 30 min. The interviews process was concluded once data saturation of results was observed [36]. Saturation is achieved when there is enough information, no more capacity to obtain new information, and additional coding is no longer possible. To achieve data saturation, we used a saturation grid, cataloging the main topics stated per interviewee [37]. Additionally, several works on related topics have followed similar strategy with similar number of interviewees [34,35].

To analyze the qualitative data, we employed an open coding methodology. The process consisted in transcribing all interviews, retrieving a list of codes and respective quotes from these transcriptions to represent the main topics referred, and finally grouping the codes into categories of similar meaning [1]. In this section we present the results from the interviews, following this methodology.

### 3.2 Qualitative study results

Throughout the interviews, interviewees highlighted the importance of knowledge regarding DTs when it comes to the adoption of the technology. Participants also expressed major concern when enquired about the possible risks to their personal privacy. These aspects were grouped into the informational factors category, as can be observed in Table 2.

**Table 2:** Informational factors quotes.

Informational Factors Quotes	
Knowledge	
I1	“It's an impediment since we don't know much about the technology you know.”
I7	“I think it's the reason for older people not to adopt a digital twin might be that they are not really familiar with using technology, so they are more afraid to not be able to control it and maybe it's more difficult for them to learn.”
I9	“So, I think that information is definitely a point of improvement because if you don't know it exists, you're not going to use it.”
I11	“I also don't know how the heating system in my building works or how the electricity behind my light switch is wired. I've no idea, and I think the same would be about a well-designed (…) digital twin system for energy, that as long as I don't need to fix it myself and as long as its running without me noticing, I don't need to know much about it.”
I14	“(…) at least I think that, and at least in our studies, that we done before the information is very, very important driver for the people use. In other words, if the people don't have information about these solutions will not use. I think that the information is very, very useful. And very important. ”
Perceived risks to personal privacy	
I1	“If it is really necessary I would go along with it, but I'm not pleased.”
I3	“My concerns about the digital twin it's privacy.”
I4	“I don't trust very much sharing my personal data, I don't like it.”
I5	“To adopt a digital twin, I would need to be comfortable with the company that is offering me the service.”
I7	“I think that a lot of people, especially older people, are still not sufficiently informed about how privacy plays on the adoption of digital twins so they might be concerned that their house can be controlled.”
I9	“(…) with social media accounts being hacked, you don't want to bring that world into your household, it's your little shelter. And I think that, that would be my main concern in terms of adopting a digital twin. And I think that I would only do so if I knew that there was privacy control and that it was safe both for me and for the people that live with me essentially.”
I13	“(…) if you have a digital twin, you also have a door for everybody to enter in your house. So it has to be constructed in a way that can assure you that it's completely safe. And that's a concern. I think it's essentially the major concern.”

During the interviews, the environmental topic was constantly present. Most interviewees displayed a great concern regarding the environment and had a positive attitude towards sustainable technologies and energy. These elements are represented as the environmental factors category, as can be seen in Table 3.

**Table 3:** Environmental factors quotes.

Environmental Factors Quotes	
Green self-identity	
I2	“(…) of course I would go for a digital twin if I was explained before that it was a good way to save the environment”
I6	“Well, the part where you can help save the environment and basically reduce energy waste that's for sure the main points.”
I8	“Specifically, I don't use for example air conditioners at home because they are not good for the environment.” (…) “I think it's actually one of the most important aspects of adopting a digital twin. Nowadays I think especially younger generations are very concerned about the environment and if we're going to adopt a new technology I think it's very important to understand if it actually is good to the environment or not.”
I9	“(…) the environmental reasons would play a big part in adopting one.”
I11	“Would allow me to clear my conscience regarding energy consumption”
I14	“Yeah, this sustainable behavior improves my well-being.”
I15	“Yes, so I believe that people that are more willing to use this type of solutions are the ones that are more concerned also about the environment.”

Throughout the interviews, participants highlighted the perceived advantages DTs would bring. These considerations were grouped into the utility factors category, as can be seen in Table 4.

**Table 4:** Utility factors quotes.

Utility Factors Quotes	
Perceived value	
I3	“I can control energy consumption; I can save money and reduce environmental impact.”
I8	“I think that in general control energy consumption will be probably one of the most important aspects due to the fact that it's the basis of a digital twin and it will be great to not only save energy but also money.”
I9	“I think that one of the biggest advantages of having, adopting a digital twin would be being able to be the most efficient in terms of your energy usage.”
I13	“(…) the opportunity of regulating the light, the heating, everything in our house is something that I think it would be useful. It would be essentially useful for your comfort. But it would be also something to save essentially, and that's why it's such a useful application.”
I14	“I think that digital twin will be very helpful to me to understand the consumptions that I have and try and have better performance in terms of less energy consumptions with the same comfort.”

Moreover, social influence was highly mentioned during interviews, especially when it comes to engaging more people in adopting DTs. This factor was organized as can be seen in Table 5.

**Table 5:** Social factors quotes.

Social Factors Quotes	
Social influence	
I2	“Firstly, they need to have like a close example, someone they know well that is using a digital twin and I think that peer-to-peer interaction is the best way to install something new on one's phone and in one's house. By having a friend or a family member that uses it and is basically familiar with that and by showing the examples of people you know that have this sort of things in their phones and houses I think it's the best way to engage with more public, who could be sceptical or simply don't be very familiar with this.”
I6	“(…) probably hearing about it more or more people with experience using digital twins would help me wanting to adopt a digital twin.”
I10	“I think by the word of mouth, like recommending it to our friends and family.”
I14	“(…) and I think that also can be a question of status (…) if the others are using and this is trendy and maybe I'll also use.”
I15	“And maybe in something that we also saw more or less in some other studies is that if people have the possibility to see with their eyes, or to try, or to have a demonstration, maybe it can help people to understand that it's easy to use.”

Finally, various interviewees highlighted the importance of comfort when it comes to the use of DTs. Quotes regarding this topic can be seen in Table 6.

**Table 6:** Comfort sensation quotes.

Comfort Sensation Quotes	
I6	“(…) just how easy it would be to set things to your liking.”
I8	“I think that digital twins can make your life more comfortable.”
I12	“(…) eventually have a more, a nicer, temperature in the house. Reducing noise in case the machines work at night or at some hour where I'm not at home.”
I13	“Well, if it allows you to save and to be more comfortable in your house. I think it's a good combination.”
I15	“Because at the end, of course we want to save energy and so on. But it is about having our houses comfortable so we can have quality on the house. ”

## 4 Research model

**Fig. 1:**
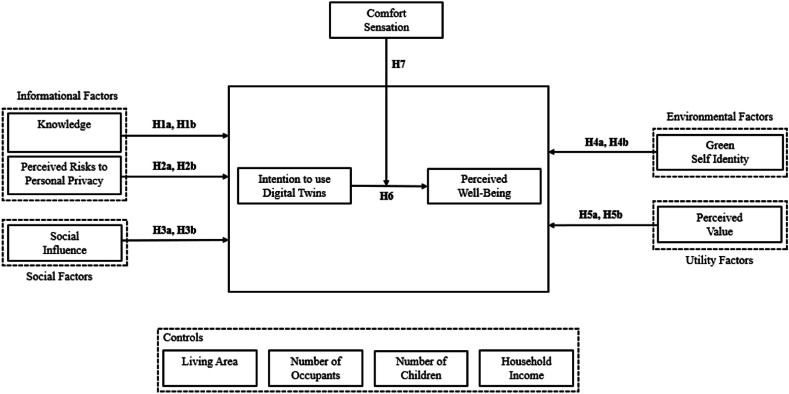
Research model. Note: H – Hypothesis.

The proposed research model (Fig. 1) is based on the literature and interviews conducted. Significant aspects referred in the interviews, combined with theoretical knowledge allowed to develop a more complete research model. From this research, we identified four main factors that influence the adoption of DTs. These factors consist of: (1) informational factors: representing the knowledge about DTs and englobing the perceived privacy risks associated with its' use; (2) social factors: representing the power of social influence; (3) environmental factors: representing the consumers’ standpoint towards the environment and sustainability and (4) utility factors: representing the perceived utility, operation needs and advantages associated to DTs. As dependent variables, we rely on the intention to use DTs and the perceived well-being enabled by DTs, given that these can be determinants of consumer behavior.

### 4.1 Explanatory variables

Due to DT being an emerging and recent technology, knowledge about it among consumers is still on an early stage. Moreover, since the application of DTs relies on the retrieval and analysis of data through other technologies [9,19], privacy concerns are raised about its deployment [28]. Thus, we present the following informational factors in our model - knowledge [38] and perceived risks to personal privacy [39].

Knowledge relates to the amount of information an individual pertains relating to DTs. The more a consumer knows about a product, the more likely he is to use it, which also applies to green energies [28]. Although environmental knowledge positively affects the intention to adopt green products [38], information about DTs is not widely disseminated yet, and several people are not familiar with the concept. Thus, we present the following hypothesis:

H1a Knowledge positively influence the intention to use DTs.

H1b Knowledge positively influence the perceived well-being enabled by DTs.

Perceived risks to personal privacy consist of the consumers’ concern about the misuse of personal data. Privacy is one of the main concerns of consumers when it comes to the adoption of smart and environmental technologies [40,41]. Thus, worries about information privacy can lead consumers to hold back when it comes to trusting businesses with their data [39] and negatively affect the adoption of sustainable energy solutions [1,28]. Hence, we elaborate the following hypothesis:

H2 Perceived risks to personal privacy negatively influence **a)** the intention to use DTs; **b)** the perceived well-being enabled by DTs.

Additionally, Social influence has a major role in green adoption, since green consumption behavior is also a socially responsible behavior [42]. All consumers are part of a social group, and are likely to follow this group when it comes to the adoption of green practices [25]. Social influence is also proven to play a significant role in the adoption of new technologies [43]. Accordingly, we raise the subsequent hypothesis:

H3 Social influence positively influence **a)** the intention to use DTs; **b)** the perceived well-being enabled by DTs.

Regarding environmental factors, motivation is a strong predictor of the adoption of technologies that enable sustainability in private households [1]. Being DTs a sustainable energy solution, in the model we portray this motivation as environmental factors, namely green self identity [44].

Green self identity relates to the level of environmental concern of an individual [31]. Correspondingly, environmental concern plays a significant part in the adoption of green energy [29], and has a positive effect on the attitude towards the adoption of eco-friendly products [44]. Thus, the hypothesis presented are:

H4 Green self-identity positively influence **a)** the intention to use DTs; **b)** the perceived well-being enabled by DTs.

Finally, to adopt any technology, a consumer evaluates its’ benefits and function according to initial needs. Thus, this model presents utility factors as drivers to the adoption of DTs, which consist of perceived value [45].

Perceived value represents the perceived advantages that DTs have for a user. The users' perceived value of a product is a major determinant of adoption intention [45], as if this perception is positive, it will have a positive impact on adoption. Moreover, perceived value of green electricity is positively affected by its’ perceived benefits [46]. Accordingly, we raise the subsequent hypothesis:

H5 Perceived value positively influence **a)** the intention to use DTs; **b)** the perceived well-being enabled by DTs.

### 4.2 Dependent variables

Overall, most studies have focused on the impact of technology in more practical or technical outcomes, instead of focusing on humanistic ones [47]. Therefore, in an attempt to reinforce this humanistic perspective, we decided to analyze the impact of DTs on the perceived wellbeing. Many times, the use of these type of solutions implies an activity to pursue a purpose (e.g. savings, cost reduction, environmental protection, among others). Therefore, when a consumer uses a solution that allows him/her to be a step closer to those goals, he/she starts to feel some sort of tranquility and achievement, engaging in a state of well-being. This is aligned with prior studies, finding that adopting pro-environmental behaviors positively impacts the state of well-being [34]. Perceived well-being relates to the level to which a DT can improve the daily life of the consumer. Customers’ well-being increases when a product has better functionality, convenience, safety, leisure, atmospherics, and self-identification [48], making this variable dependent of the identified adoption factors. Therefore, we elaborate the following hypothesis:

H6 Intention to use DTs positively influence perceived well-being enabled by adopting a DT.

### 4.3 Moderator variable

Comfort sensation entails the perceived comfort resultant from using a DT. Literature suggests that by employing a DT, the user will have a more comfortable experience [19]. In the energy consumption scenario, DTs have the power to regulate room temperature to a comfortable setting [21], without overusing heating or cooling equipments. Hence, we hypothesize:

H7 Comfort sensation moderates the perceived well-being enabled by adopting a DT.

### 4.4 Controls

When it comes to the adoption of new technologies, consumer behavior is usually controlled by socio-demographic factors. In the case of the adoption of energy-related technologies, it is relevant to include controls related to the household itself [30,31,49,50]. The living area, number of occupants, number of occupants under 18 years old and household income are therefore the control variables of our model.

## 5 Quantitative study

### 5.1 Methodology

To validate the model's hypothesis, we relied on the implementation of an online questionnaire. To better fit this study's context, each construct's item was adapted from literature regarding the adoption of technologies and sustainable energy systems. In most questions, respondents were asked to rate their agreement with each item statement on a seven-point numerical scale (1 – completely disagree and 7 – completely agree). To facilitate its dissemination, the questionnaire was elaborated in English and distributed online through social media platforms. As we are studying the household adoption of a new technology, a questionnaire filter was employed, so that only respondents who participate in the decision-making process of their household would be able to undertake it. Preceding the questions, the survey included an introductory text and image explaining the DT concept. We undertook a pilot study with 30 responses, which yielded positive results regarding the validity and reliability of the questionnaire. Table 7 presents the constructs and items used to build the survey.

**Table 7:** Table of constructs.

Construct	Items	Reference
Independent variables		
Knowledge	K1. I am knowledgeable about energy topic and the environment (dropped)	[38]
	K2. I am familiar with the Digital Twin concept	
	K3. I know how to adopt a Digital Twin	
Perceived risks to personal privacy	PR1. Digital Twins would collect too much information about me	[39]
	PR2. I would be concerned about my privacy when using Digital Twins	
	PR3. All things considered, a Digital Twin would cause serious privacy problems	
	PR4. My personal information would be misused when using Digital Twins	
	PR5. My personal information would be accessed by unknown parties when using Digital Twins in my everyday life	
Green self identity	GS1. I consider myself worried with environmental problems	[44]
	GS2. I consider myself a “green consumer"	
	GS3. I worry about the effects of energy consumption on the environment	
	GS4. I worry about atmospheric pollution caused by the energy consumption	
Perceived value	PV1. Compared to the effort I need to put in, the use of a Digital Twin is beneficial to me	[45]
	PV2. Compared to the time I need to spend, the use of a Digital Twin is worthwhile to me	
	PV3. Overall, the use of a Digital Twin delivers good value for me	
Social influence	SI1. People who are important to me, would think that I should adopt a Digital Twin	[43]
	SI2. People who influence my behavior, would think that I should adopt a Digital Twin	
	SI3. People who are in my social circle, would think that I should adopt a Digital Twin	
	OM2. I believe that a Digital Twin needs the user to perform maintenance work by himself (dropped)	
	OM3. I believe that the maintenance of a Digital Twin requires too much work	
Comfort sensation	CS1. Visual comfort (with aspects such as view, illuminance, and reflection) (dropped)	[51]
	CS2. Thermal comfort in heating season (air velocity, humidity, and temperature) (dropped)	
	CS3. Thermal comfort in cooling season (air velocity, humidity, and temperature)	
	CS4. Acoustical comfort (control of unwanted noise, vibrations, and reverberations)	
	CS5. Air quality (smells, irritants, outdoor air, and ventilation)	
Dependent variables		
Intention to use Digital Twins	IU1. I intend to adopt a Digital Twin in the future	[43]
	IU2. I will try to adopt a Digital Twin in the future	
	IU3. I am ready to adopt a Digital Twin	
Perceived wellbeing	PW1. Satisfy my overall household needs	[48]
	PW2. Play a very important role in my social well-being	
	PW3. Play a very important role in my leisure well-being	
	PW4. Play an important role in enhancing the quality of life in my household	

### 5.2 Sample and data

Common method bias was also evaluated. Using Harman's one-factor test [52] we have concluded that the first factor explains 32,97 % of variance, meaning that none of the factors individually explain variance on more than 50 %. Afterwards, using the marker variable method [52], we obtained a maximum shared variance with other values of 0.06 (6 %). Therefore, no significant common method bias was discovered. A calculation for the minimum sample size was performed using the sample size formula for an infinite population, specifically (n = Z2p * q/d2), where Z is the standard normal distribution for the (1 − α/2) level, d is the precision, p is the prevalence, and q = (1 − p). Regarding the prevalence (p), we have resorted to statistical data on the adoption of IoT in Portugal households, showing a 9.3 % penetration rate in households [53]. A level of precision (d) of 5 % was also assumed. Given this, the minimum sample size needed was 130 respondents. The questionnaire was disseminated online through social media tools and collected anonymous data from 182 respondents from Portugal. From these, only 149 respondents fully completed the questionnaire due to the implemented filter. Table 8 characterizes the sample data regarding living area, household monthly net income, number of household habitants and number of household habitants under 18 years old.

**Table 8:** Descriptive statistics of the sample.

Sample characteristics (n = 149)	Descriptive statistics
**Living Area**	
Rural	20,13 %
Urban	79,87 %
**Household monthly net income**	
Bellow 2500€	53,02 %
Above 2500€	46,98 %
**Number of household habitants**	
One to four	82,55 %
More than four	17,45 %
**Number of household habitants under 18 years old**	
Zero	70,47 %
More than zero	29,53 %

## 6 Results

To estimate the model, we used the partial least squares (PLS) technique. This method supports the goal of this study of identifying key driver constructs and admits a complex structural model with a small sample size [54]. Therefore, PLS SEM allows to test the proposed hypothesis, evaluating the relationships between constructs. SmartPLS 4.0 was the chosen software to analyze the model and its results [55]. To validate the model, first the measurement will be assessed, followed by the structural model.

### 6.1 Measurement model

To assess the measurement model, we analyzed different measures for the reflective constructs (Table 9). For these, we determined the descriptive statistics of the mean and standard deviation, inferred the composite reliability, determined the indicator reliability, evaluated the convergent validity and assessed the discriminant validity. Considering Cronbach's alpha, all constructs present values above 0.708, and an AVE higher than 0.5, thus confirming internal consistency reliability and convergent validity [54,56]. Indicator reliability was also ensured since all the indicator's outer loadings are higher than 0.708 (**Appendix C**). This indicates that our constructs are reliable and consistent. Discriminant validity of the reflective constructs was assessed through the Heterotrait-Monotrait Ratio (HTMT), Fornell-Larcker criterion, and cross-loadings. Since all values in the HTMT (**Appendix D**) are lower than 0.9, the square root of AVE of each construct is higher than its highest correlation with other constructs (Table 9), and the indicator's outer loadings on their construct are higher than all its cross loadings with other constructs (**Appendix C**), discriminant validity of the reflective constructs is ensured [56]. This indicates that there is no overlapping between constructs, and therefore each factor is measuring solely what is intended. Given, this the structural model can now be evaluated.

**Table 9:** Mean, Standard-Deviation (SD), Composite Reliability (CR), and Fornell-Larcker table.

Construct	Mean	SD	CR	CS	GS	IU	K	PR	PV	PW	SI
Comfort sensation (CS)	5,17	1,56	0.740	**0.807**							
Green self identity (GS)	5,66	1,42	0.885	0.058	**0.863**						
Intention to use Digital Twins (IU)	4,62	1,81	0.830	0.180	0.368	**0.867**					
Knowledge (K)	3,58	1,98	0.891	0.159	0.265	0.529	**0.949**				
Perceived risks to personal privacy (PR)	4,06	1,86	0.907	−0.023	−0.005	−0.235	−0.052	**0.850**			
Perceived value (PV)	4,98	1,44	0.932	0.274	0.361	0.719	0.487	−0.120	**0.938**		
Perceived wellbeing (PW)	4,61	1,74	0.913	0.186	0.227	0.711	0.362	−0.105	0.705	**0.890**	
Social influence (SI)	4,26	1,73	0.917	0.234	0.243	0.691	0.462	−0.146	0.646	0.678	**0.927**

**Note:** The diagonal elements are the square-root of AVE.

### 6.2 Structural model

**Fig. 2:**
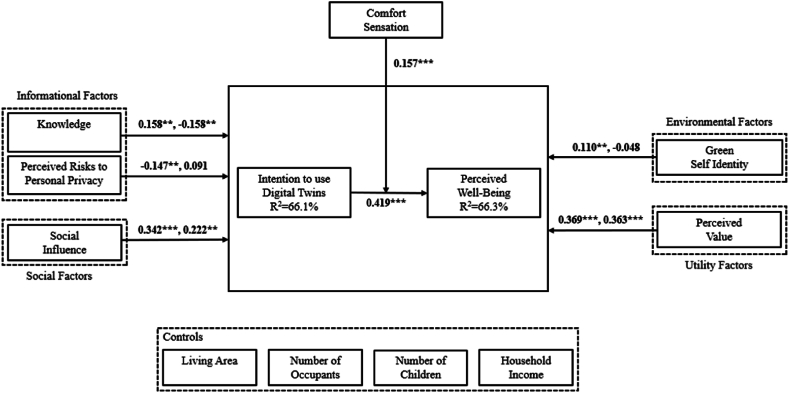
Structural model of the intention to use DT. **Notes:** ***p < 0.01; **p < 0.05; *p < 0.1.

Before analyzing the structural model, we have to assess multicollinearity, we analyzed the VIF values of all constructs. The highest VIF value observed is 3.014, demonstrating that all values are below 5, which proves the inexistence of collinearity issues, and therefore no explanatory constructs variance are being explained by other explanatory ones that may lead to bias relationships [54]. The structural model presented in Fig. 2 demonstrates the path coefficients and variance proportion (R^2^). We used the bootstrapping method with 5000 resamples to assess the significance of the constructs of the proposed model.

This model explains in 66.1 % the intention to use DTs and in 66.3 % the perceived well-being from using a DT. The knowledge construct is statistically significant for the perceived well-being and intention to use DT ($\hat{\beta }a$ = 0.158, p < 0.05; $\hat{\beta }b$ = −0.158, p < 0.05). Thus, H1a and H1b are supported. The perceived risks to personal privacy construct is statistically significant only for the intention to use DT ($\hat{\beta }a$ = −0.147, p < 0.05). Thus, H2a is confirmed but H2b is not. The social influence construct is statistically significant for both the perceived well-being and intention to use DT ($\hat{\beta }a$ = 0.342, p < 0.01; $\hat{\beta }b$ = 0.222, p < 0.05). Thus, H3a and H3b are confirmed. The green self identity construct is statistically significant for the intention to use DT ($\hat{\beta }a$ = 0.110, p < 0.1) but not for the perceived well-being. Thus, H4a is supported but H4b is not. The perceived value construct is statistically significant for both the perceived well-being and intention to use DT ($\hat{\beta }a$ = 0.369, p < 0.01; $\hat{\beta }b$ = 0.363, p < 0.01). Thus, H5a and H5b are supported. The intention to use DT construct is statistically significant for the perceived well-being ($\hat{\beta }$ = 0.419, p < 0.01). Thus, H6 is confirmed. Finally, the comfort sensation construct is statistically significant as a moderator between the intention to use DT and perceived well-being ($\hat{\beta }$ = 0.157, p < 0.01). Thus, H7 is validated. Consequently, out of the 12 proposed hypothesis in our research model, 10 are supported, being that all hypothesis related to the adoption of DTs are confirmed.

## 7 Discussion

This study contributes to the existing literature on both DTs and the adoption of new technologies, as it proposes a model of the adoption of DTs in households related to the we-being enabled by this adoption. Our model explains a set of factors that lead to the adoption of DT, which can help identify key consumer preferences. Previous literature has focused on the drivers of adoption of other sustainable energy solutions [1,[28], [29], [30], [31]]. Our study complements these by doing an in-depth analysis of the adoption of DTs specifically, determining that the adoption of this technology has some drivers in common with previous studies. The proposed structural model contributes to the advancement of DTs research in the context of households. Findings can also be applied when studying the adoption of similar technologies. Therefore, this can be seen as a basis framework to foster future studies on the consumer perspective of digital twin technology.

The developed model of the adoption of DTs in households can have several practical implications. The proposed model allows for the improved understanding of consumer behavior, as it introduces the DT's adoption drivers and barriers, which can be explored as a marketing strategy. Moreover, this model presents an opportunity to optimize DT development regarding its' design and functionality, to meet consumer's needs. Finally, model insights provide an opportunity for increasing the adoption of DTs, as consumer concerns and barriers to adoption can be minimized.

This paper concludes that a set of informational, social, environmental and utility factors have a strong impact on a consumer's decision to adopt a DT. These factors explain in 66.1 % the consumer's intention to use a DT. Since knowledge regarding the technology itself has a positive impact on the adoption intention, it is crucial to spread information regarding DTs, namely its availability, how it can be used and what are the advantages associated to it. It was also discovered that social influence plays a major role not only in the intention to use DT but also in the perceived well-being from DT usage. Therefore, positive word-of-mouth and marketing strategies such as the use of social media influencers can be beneficial. Green self identity was also considered valuable for the adoption of DTs, thus disseminating information on the sustainability impact of a DT is likely to attract environmentally concerned consumers to the product. The perceived value and advantages from using a DT also impact positively both the perceived well-being and use intention of DTs. Thus, providers should invest in promoting the effortless benefits a DT can bring into households. Moreover, the use of a DT moderated by the comfort sensation it enables contributes to the perceived well-being of a consumer. Accordingly, it is important to disseminate how a DT can improve the user's quality of living by causing homes to become more comfortable. Additionally, as initially expected, at a time when privacy has proven to negatively impact user's satisfaction regarding technologies [57], perceived risks to personal privacy act as a barrier to DT adoption. Thus, companies must ensure that all data collected will be stored, treated and used according to all regulations in place, i.e. GDPR in the EU [58]. Likewise, knowledge acts as a barrier when it comes to the perceived well-being enabled by DTs. Lack of widespread information regarding the technology does not limit the consumers' intention to adopt DTs but impacts perceived well-being as the adoption process is unknown and users are not familiar with the technology. Thus, information regarding this topic should be widely disseminated in a simple manner, decreasing any concerns that may arise.

The proposed model of the adoption of DTs in households can provide useful insights and guide future research in the area. However, this model is based on assumptions and might not always reflect real-life behavior accurately. Out of the 12 proposed hypothesis in our research model, 10 are supported by this study. It could prove interesting to further explore more factors behind the perceived well-being associated with the use of DTs. Moreover, extended versions of this model or confirmation of the unsupported hypothesis could be obtained through acquiring a larger sample of observations. It could be also relevant to develop further research on the effect of other moderator variables in the model, being that consumer behavior is usually controlled by socio-demographic factors.

## 8 Conclusion

As climate change has become our somber reality, adoption of sustainable energy solutions among a broad public has become a priority. It is crucial to understand consumer behavior when it comes to this topic, namely what exactly are the factors pushing back from a widespread adoption. The adoption of DTs in households could help mitigating energetic crisis to a certain point. This study contributes to better understand the drivers that lead to the adoption of DT. From a more theoretical perspective, this is one of the first articles that explore the DTs adoption from the households/consumers point of view, shedding light on the main motivators and concerns of individuals when adopting DTs technology. By identifying the main determinants of adoption, it was possible to develop recommendations to increase the adoption of DT technology. Moreover, this article establishes the perceived impact of DTs on well-being, contributing to a more humanistic study of technology impacts. By establishing the strong positive relationship between the adoption of DT and perceived well-being, we recognize the impact technologies can have on users' humanistic outcomes, such as satisfaction of needs and quality of life, contributing to a more humanized view of technology, instead of focusing on more instrumental outcomes, as productivity, efficiency, etc. In doing so, we conclude that the adoption of DT can in fact be also seen as an investment in users’ quality of life and therefore increase its diffusion. From a practical point of view, this work allows to better understand consumer behavior and thus employ planned marketing and policy strategies to better target the public on adopting DTs technology. Findings confirmed that knowledge, social influence, green self identity, perceived value and comfort sensation have a positive influence on intention to use DTs and its consequent perceived well-being. In contrast, the perceived risks to personal privacy and knowledge might act as barriers to adoption and well-being respectively. Therefore, by identifying these motivations, it is possible to better support strategies to boost the implementation of DTs for sustainable purposes.

## Ethics statement

All participants provided informed consent to participate in the study.

## Data availability statement

Data will be available on request from the corresponding author.

## Additional information

No additional information is available for this paper.

## CRediT authorship contribution statement

**Joana Coelho:** Writing – original draft, Software, Methodology, Investigation, Formal analysis, Conceptualization. **Tiago Oliveira:** Writing – review & editing, Validation, Supervision, Methodology, Conceptualization. **Catarina Neves:** Writing – review & editing, Validation, Supervision, Methodology, Conceptualization. **Stylianos Karatzas:** Validation, Supervision, Investigation, Conceptualization.

## Declaration of competing interest

The authors declare that they have no known competing financial interests or personal relationships that could have appeared to influence the work reported in this paper.

## Figures and Tables

**Table:** 

	Role	Occupation	Household Role	Household Type	Potential Adopter?
**#**	Consumer	Teacher	Decision-maker	Flat	Yes
I2	Consumer	Public worker at a European institution	Decision-maker	Flat	Yes
I3	Consumer	Accountant Junior	Decision-maker	Detached house	Yes
I4	Consumer	Judge at the court of appeal	Decision-maker	Flat	Yes
I5	Consumer	Controlling and reporting trainee	Decision-maker	Flat	Yes
I6	Consumer	Student	Decision-maker	Detached house	Yes
I7	Consumer	Product insights and analytics intern	Decision-maker	Flat	Yes
I8	Consumer	Student	Not the decision-maker	Semidetached house	Yes
I9	Consumer	Film, TV and media producer	Decision-maker	Flat and detached house	Yes
I10	Consumer	Student	Not the decision-maker	Flat	Yes
I11	Consumer	Student	Not the decision-maker	Flat	Yes
I12	Consumer	Professor	Decision-maker	Flat	Yes
I13	Consumer	Judge	Not the decision-maker	Flat	Yes
I14	Expert	Professor	Decision-maker	Flat	Yes
I15	Expert	Professor	Not the decision-maker	Flat	Yes

**Table:** 

Items	Comfort sensation (CS)	Green self identity (GS)	Intention to use Digital Twins (IU)	Knowledge (K)	Perceived risks to personal privacy (PR)	Perceived value (PV)	Perceived wellbeing (PW)	Social influence (SI)
CS3	**0.842**	0.093	0.192	0.198	−0.034	0.232	0.175	0.174
CS4	**0.833**	−0.019	0.143	0.140	−0.028	0.240	0.158	0.244
CS5	**0.744**	0.069	0.076	−0.003	0.021	0.184	0.102	0.135
GS1	0.050	**0.883**	0.315	0.201	−0.044	0.302	0.183	0.165
GS2	−0.033	**0.812**	0.322	0.279	0.011	0.296	0.248	0.285
GS3	0.133	**0.839**	0.304	0.135	−0.022	0.340	0.119	0.176
GS4	0.066	**0.915**	0.325	0.280	0.030	0.311	0.214	0.203
IU1	0.184	0.369	**0.937**	0.472	−0.215	0.744	0.703	0.674
IU2	0.175	0.351	**0.908**	0.488	−0.223	0.623	0.615	0.582
IU3	0.099	0.220	**0.743**	0.415	−0.170	0.476	0.515	0.534
K2	0.138	0.282	0.560	**0.962**	−0.051	0.505	0.374	0.479
K3	0.169	0.215	0.429	**0.936**	−0.048	0.410	0.306	0.388
PR1	0.102	−0.016	−0.094	−0.001	**0.737**	−0.003	−0.025	−0.050
PR2	−0.064	0.055	−0.186	0.011	**0.860**	−0.117	−0.055	−0.089
PR3	0.002	−0.003	−0.217	−0.071	**0.876**	−0.126	−0.078	−0.155
PR4	−0.028	−0.005	−0.172	−0.046	**0.875**	−0.069	−0.073	−0.118
PR5	−0.041	−0.041	−0.258	−0.075	**0.894**	−0.131	−0.154	−0.158
PV1	0.300	0.346	0.676	0.411	−0.162	**0.936**	0.638	0.605
PV2	0.244	0.323	0.658	0.476	−0.014	**0.944**	0.682	0.585
PV3	0.229	0.346	0.691	0.483	−0.160	**0.934**	0.663	0.627
PW1	0.170	0.294	0.669	0.354	−0.069	0.675	**0.882**	0.616
PW2	0.191	0.177	0.712	0.351	−0.165	0.636	**0.896**	0.608
PW3	0.166	0.184	0.582	0.275	−0.019	0.570	**0.894**	0.626
PW4	0.133	0.146	0.557	0.304	−0.114	0.625	**0.888**	0.562
SI1	0.247	0.221	0.602	0.407	−0.095	0.553	0.603	**0.892**
SI2	0.182	0.228	0.711	0.452	−0.184	0.641	0.644	**0.954**
SI3	0.225	0.228	0.605	0.424	−0.122	0.598	0.637	**0.934**

**Table:** 

Items	CS	GS	IU	K	PR	PV	PW	SI
Comfort Sensation (CS)								
Green self identity (GS)	0.128							
Intention to use Digital Twins (IU)	0.211	0.421						
Knowledge (K)	0.181	0.287	0.607					
Perceived risks to personal privacy (PR)	0.079	0.068	0.252	0.053				
Perceived value (PV)	0.325	0.399	0.808	0.528	0.132			
Perceived wellbeing (PW)	0.217	0.245	0.808	0.395	0.109	0.763		
Social influence (SI)	0.278	0.267	0.789	0.504	0.147	0.697	0.740	

## Appendix B Interview Guide

1. Have you ever used a Digital Twin?
a. If yes, can you report on your experience?
2. Which aspects in Digital Twins do you like? Which don't you like?
3. Which motives would be relevant to you in adopting a Digital Twin?
a. Which role do financially oriented reasons play in the adoption of a DT?
b. Which role do environmental reasons play in the adoption of a DT?
c. Which role does DT utility and advantages (eg. Control energy consumption, monetary savings, reduce environmental impact …) play in the adoption of a DT?
d. Which role do privacy concerns play in the adoption of a DT?
e. Which role does knowledge about the technology play in the adoption of a DT?
4. Which needs could you fulfill by using a DT?
5. What are your concerns regarding using a DT?
6. How would/do you use a DT?
7. Do you think information about Digital Twins is easily accessible?
8. How can more people engage in adopting Digital Twins?
9. Which aspect would have to be improved for you to adopt a Digital Twin?
10. If you could, would you adopt a Digital Twin? (if not yet adopted)
